# Advancement in research and therapy of *NF1* mutant malignant tumors

**DOI:** 10.1186/s12935-020-01570-8

**Published:** 2020-10-09

**Authors:** Junyan Tao, Dantong Sun, Lina Dong, Hua Zhu, Helei Hou

**Affiliations:** Precision Medicine Center of Oncology, the Affiliated Hospital of Qingdao University, No. 59 Haier Road, Qingdao, Shandong 266000 China

**Keywords:** *NF1*, *Ras*, Therapeutic resistance, Molecular mechanism, Malignancies

## Abstract

The *NF1* gene encodes neurofibromin, which is one of the primary negative regulatory factors of the Ras protein. Neurofibromin stimulates the GTPase activity of Ras to convert it from an active GTP-bound form to its inactive GDP-bound form through its GTPase activating protein-related domain (GRD). Therefore, neurofibromin serves as a shutdown signal for all vertebrate RAS GTPases. *NF1* mutations cause a resultant decrease in neurofibromin expression, which has been detected in many human malignancies, including NSCLC, breast cancer and so on. *NF1* mutations are associated with the underlying mechanisms of treatment resistance discovered in multiple malignancies. This paper reviews the possible mechanisms of *NF1* mutation-induced therapeutic resistance to chemotherapy, endocrine therapy and targeted therapy in malignancies. Then, we further discuss advancements in targeted therapy for *NF1*-mutated malignant tumors. In addition, therapies targeting the downstream molecules of *NF1* might be potential novel strategies for the treatment of advanced malignancies.

## Background

With the aging population and changes in lifestyle, the morbidity and mortality rates of malignancies in the world are rising drastically. In 2018, 18.1 million new cases and 9.6 million cancer deaths were registered worldwide [1]. Treatments for advanced malignant tumors, including traditional treatment (surgery combined with radiotherapy and chemotherapy), targeted therapy and immunotherapy, significantly prolong the survival of patients with malignancies. To date, targeted therapies have achieved significant advances. The studies on the driven mutations of malignancies, such as epidermal growth factor receptor (*EGFR*), anaplastic lymphoma kinase (*ALK*), ROS proto-oncogene 1 (*ROS1*) and human epidermal growth factor receptor-2 (*HER2*), have brought clinical benefits and more therapeutic options for patients with malignancies [2–4]. Patients harboring sensitive mutations benefit from targeted therapy. However, drug resistance remains a serious problem during treatment. Drug resistance limits the use of targeted therapy in malignant tumors and is one of the foremost challenges in malignant tumors today [5].

**Fig. 1:**
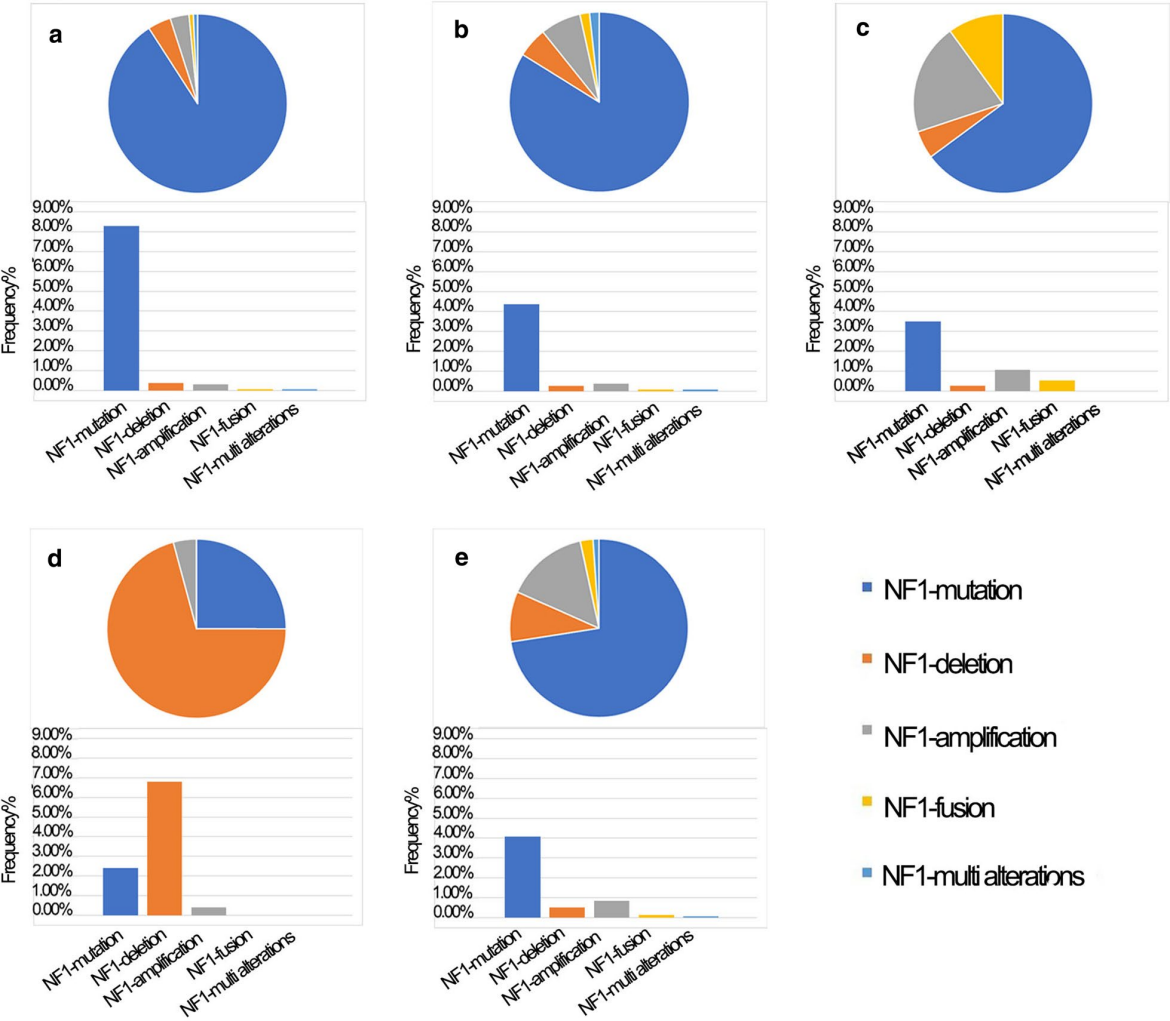
The types and frequencies of NF1 alterations in different malignancies (data cited from cBioPortal for Cancer Genomics). **a** NSCLC; **b** esophagogastric cancer; **c** liver cancer; **d** ovarian cancer; **e** breast cancer

*Neurofibromin 1* (*NF1*) mutations cause an autosomal dominant genetic susceptibility syndrome known as neurofibromatosis type 1 [6]. Furthermore, genomic data from the cBioPortal for Cancer Genomics datasets indicate that somatic *NF1* mutations can be detected in a variety of malignancies, including non-small-cell lung cancer (NSCLC), ovarian cancer, breast cancer, liver cancer, and esophagogastric cancer. The alterations and frequencies of *NF1* in malignancies are shown in Fig. 1. According to previous studies, *NF1* mutations have been detected in patients with primary and acquired resistance to tyrosine kinase inhibitors (TKIs) [7, 8]. In this case, we conclude the latest advancement in targeted therapy of malignant tumors with *NF* 1 mutations and further clarify the role of *NF1* mutations in the treatment of malignancies to establish the viability of the treatment targeting *NF1* mutations.

### *NF1* gene and neurofibromin

The *NF1* gene was initially discovered as a tumor suppressor in the early 1990s [9–12]. It is located on the long arm 11.2 of chromosome 17 (17q11.2). The types of *NF1* mutations are diverse, including missense/nonsense (27.7%), microdeletions (26.9%), gross deletions (> 20 bp; 13.3%), splicing (16.3%), microinsertions (11.1%), indels (2.0%), gross insertions (> 20 bp; 2.0%), complex rearrangements (0.6%) and several putative regulatory mutations [13]. Neurofibromin, encoded by the *NF1* gene, is a large multi-domain 2818 amino acid protein with a molecular weight of approximately 220 kDa [14]. Neurofibromin contains several domains, including a cysteine–serine-rich domain (CSRD), a central GTPase-activating protein-related domain (GRD), a tubulin-binding domain (TBD), a SEC14 domain, a carboxy-terminal domain (CTD), a pleckstrin homology (PH) domain and a syndecan-binding domain (SBD). The different domains of the neurofibromin protein is shown in Additional file 1: Figure S1. Neurofibromin binds to GTP-bound Ras through its GRD to regulate the function of Ras. The *NF1* gene contains 60 exons and produces multiple alternative splicing isoforms [15]. Exon 23 encodes part of the GRD. An exon 23 splice variant inserts an alternative exon 23a, and exon 23a inclusion specifically decreases the Ras-GAP (GTPase activating protein) activity of neurofibromin [16]. Several mechanisms have been confirmed to be associated with the downregulation or even the loss of neurofibromin expression in tumors, such as mutations of the *NF1* gene, ubiquitin-mediated proteasomal degradation of neurofibromin [17] and promoter methylation or miRNA-mediated degradation, which inactivates the transcription of the *NF1* gene [18, 19].

## The biological function of the *NF1* gene

### The relationship between *NF1* and *Ras*

*NF1* is a tumor suppressor gene and a negative regulator of Ras protein. Under physiological conditions, neurofibromin, encoded by the *NF1* gene, stimulates the GTPase activity of Ras to convert it from an active GTP-bound form to its inactive GDP-bound form through GRD [20]. Therefore, neurofibromin can serve as a shutdown signal for all vertebrate RAS GTPases (including KRAS, NRAS, HRAS, MRAS, RRAS and RRAS2) [21]. The functional loss of neurofibromin caused by *NF1* mutations will lead to sustained activation of intracellular RAS-GTP and prolonged activation of the RAS/RAF/MAPK signaling pathway, which eventually results in increased cellular proliferation and even uncontrollable tumor growth. In addition to the loss of the function of the shutdown signal, *NF1* mutations increase the number of possible subsequent mutations, which can further upregulate Ras signaling [22]. Ras proteins regulate cell fates by cycling between active GTP–bound and GDP–bound conformations [23] and the activation of the Ras signaling pathway is one of the major driving pathways of malignancies (Table 1).

**Table 1:** The mechanisms of therapeutic resistance induced by *NF1* mutations

Alteration induced by NF1 mutations	Downstream factors	Related therapeutic resistance	References
Inhibition of cisplatin-induced apoptosis	MCL1↓	Cisplatin	[35]
Heat shock response	HSF1↑	Trastuzumab, lapatinib	[44–48]
EMT	Invasion and migration	Targeted therapy	[51–54]
mTOR-HIF-1α-VEGF pathway↑	VEGF↑	TKIs	[57–60]
Ras-dependent pathways	Ras↑	TKIs	[8, 41, 49]

“↓”means down regulation of expression; “↑”means upregulation of expression

### The relationship between *NF1* and other proteins

Mammalian target of rapamycin (mTOR), an evolutionarily conserved serine-threonine protein kinase, is a downstream effector of Ras and regulates cell proliferation and other biological behaviors [24]. Johannessen et al. demonstrated that neurofibromin was essential for moderately suppressing mTOR signaling in the absence of mitogenic stimuli. They identified *NF1* as one of the oncogenes involved in mTOR activation [25]. In mammalian cells, the mTOR complex is a complex with two forms: mTORC1 and mTORC2 [26]. Malone et al. found that mTORC1 was the key PI3K pathway component in malignancies of *NF1* mutations, while mTORC2 was dispensable [27]. In addition to Ras and mTORC1, *NF1* is associated with various protein molecules, such as focal adhesion kinase (FAK) and valosin-containing protein (VCP). Study by Tsai. et al. reveals *NF1* has relationship with integrin/FAK signaling in synapse growth modulation. Genetic and protein–protein interaction between *NF1* and FAK suggested that *NF1* functions downstream of and forms a protein complex with FAK that mediates *NF1* signaling activity and synaptic localization [28]. Wang.et al. showed that neurofibromin and VCP interact and work together to control the density of dendritic spines [29]. These studies suggested neurofibromin, encoded by the *NF1* gene, has been shown to regulate synaptogenesis. In this review, we mainly discuss the relationship between *NF1* and cancer therapeutic resistance, especially resistance to TKIs; therefore, the relationship between *NF1* and other protein molecules will not be restated.

## The role of *NF1* in anticancer therapies

### Relationship between *NF1* mutations and radiotherapy

Studies have shown that radiotherapy increased the incidence of second malignant neoplasms (SMNs) in patients with *NF1* mutations [30–32]. Unlike primary cancers, SMNs are therapy-induced malignancies and are becoming a problem that cannot be ignored in cancer survivors.

Choi et al. found that both irradiated wild-type and *NF1* mutated mice developed multiple malignancies in a dose-dependent manner in the irradiation field. However, at each radiation dose level, *NF1*-mutated mice developed more malignancies than matched wild-type mice. They then further analyzed clinical SMN samples and confirmed that among patients with radiation-induced breast cancer, the loss of constitutional heterozygosity (LOH) of *NF1* was identified in unrelated individuals without neurofibromatosis type 1 [31]. The mechanism by which *NF1* mutations promote radiation-induced SMNs is poorly understood. Several studies speculated that it might be related to the hyperactivity of the Ras signaling pathway and the loss of tumor protein p53 (*TP53)* adjacent to *NF1* on the chromosome [31, 32], but the specific mechanism needs further research.

### The role of NF1 in resistance to chemotherapy and endocrine therapy

Myeloid cell leukemia 1 (MCL1), which is an antiapoptotic protein in ovarian cancer [33], allows cancer cells to evade apoptosis when its expression is upregulated [34]. Su et al. demonstrated that the loss of NF1 observably upregulated MCL1 expression and endowed ovarian cancer cells with antiapoptotic capability through miR-142-5p. They further confirmed that NF1 loss inhibited cisplatin-induced apoptosis and resulted in resistance to chemotherapy in ovarian cancer cells [35].

Sokol et al. demonstrated that *NF1* mutations lead to tamoxifen acquired resistance in invasive lobular carcinomas (ILCs) of breast cancer. *NF1* mutations are enriched in ILCs, especially in metastatic ILCs, which exhibit a higher frequency of *NF1* mutations. Under the appropriate conditions, the loss of NF1 can enhance the competitive growth advantages of breast cancer cells harboring *NF1* mutations. Loss of NF1 was detected to cooccur with cadherin 1 (CDH1) inactivation and AKT pathway activation, and either or both of these pathway alterations may facilitate endocrine therapy resistance, which remains to be further studied [36].

## The underlying mechanisms of TKI resistance induced by *NF1* mutations

The relationship between *NF1* mutations and resistance to TKIs in malignancies has not been extensively explored. Previous studies have demonstrated that mutations in *NF1* are related to TKI resistance [8, 20, 37]. However, the mechanism of resistance to TKIs induced by *NF1* mutations remains unclear. Nevertheless, research data have suggested that treatments targeting MEK or mTOR are effective and even synergetic for malignancies with *NF1* mutations [38–40]. Clinically, mutations of *NF1* have also been confirmed to be related to resistance to other agents of targeted therapy. Retinoic acid (RA) is one of the few targeted therapies currently used in the clinic for invasive neuroblastoma. Loss of NF1 contributes to resistance to RA; moreover, the inhibition of MEK signaling downstream restores responsiveness to RA treatment [41]. Another recent clinical study revealed that low expression of NF1 is associated with more extensive lymph node metastases and poor prognosis in epithelial ovarian cancer patients [42]. According to the latest studies, the underlying mechanisms of TKI resistance induced by *NF1* mutations are summarized as follows.

### Activation of heat shock factor 1

Heat shock factor 1 (HSF1), an essential conserved master transcriptional regulator in eukaryotic cells, is critical for maintaining homeostasis of the cell proteome [43]. Studies have shown that HSF1 plays an important role in a variety of basic cellular processes essential for carcinogenesis, including cell cycle control, glucose metabolism, ribosome biogenesis, and protein translation. Genetic aberrant *HSF1* might attenuate tumorigenesis and cellular transformation driven by oncogene activation or the loss of tumor suppressors both in mice and human cell lines [44, 45]. Dai et al. found that NF1 was a potent regulator of HSF1 and changed the expression and activation of HSF1. *NF1* deficiency upregulated HSF1 and activated the heat shock response [46]. A study identified that the augmentation of the HSF1-mediated heat shock response is responsible for lapatinib resistance in breast cancer [47]. Another study showed that increased glycolysis via HSF1 contributes to trastuzumab resistance [48]. Therefore, although there is no direct verification, it is feasible that *NF1* mutations might participate in the resistance to TKIs by upregulating HSF1. This mechanism requires further study.

### Inhibition of tumor cell apoptosis

Shapira et al. confirmed that neurofibromin exerted its tumor suppressor function by enhancing the sensitivity of apoptosis via Ras-dependent pathways. In their study, the administration of farnesyl thiosalicylic acid (FTS), which is a Ras inhibitor, increased the apoptosis of neurofibromin-deficient mouse embryonic fibroblasts (MEFs) and malignant peripheral nerve sheath tumor (MPNST) cells [49]. A study provided evidence to suggest that *NF1* silencing decreased the sensitivity of erlotinib-induced cell apoptosis and/or growth arrest in lung adenocarcinoma cells. The study revealed that the reduction of neurofibromin expression increased Ras activity and weakened the effect of erlotinib on the downstream MAPK pathway, thereby decreasing the sensitivity of EGFR inhibitory drugs and eventually leading to the resultant resistance to erlotinib. Neurofibromin influenced erlotinib sensitivity through its function as a negative regulator of Ras protein [8]. Therefore, the antiapoptotic effect caused by *NF1* mutations through the Ras-dependent pathway may be one of the mechanisms of TKI resistance in malignancies.

### Promotion of epithelial-mesenchymal transformation

Epithelial-mesenchymal transition (EMT) is a process of the loss of epithelial characteristics and the acquisition of a mesenchymal phenotype, which is mediated by the activation of EMT transcription factors (EMT-TFs) [50]. The EMT process is associated with the resistance of multiple therapeutics in tumor cells by enhancing the migration and invasion of tumor cells. Tumor cells harboring the EMT phenotype showed intrinsic resistance to EGFR TKIs [51–53]. Arima et al. demonstrated that silencing *NF1* induced the expression of EMT-TFs in normal human Schwann cells and epithelioid breast cancer cells, suggesting that the loss of neurofibromin expression might activate the EMT-related signaling pathway [54]. As a result, neurofibromin might inhibit the EMT process, while *NF1* mutations, which contribute to the loss of neurofibromin expression, could be the underlying mechanism of TKI resistance in malignancies.

### Promotion of sustained angiogenesis

**Fig. 2:**
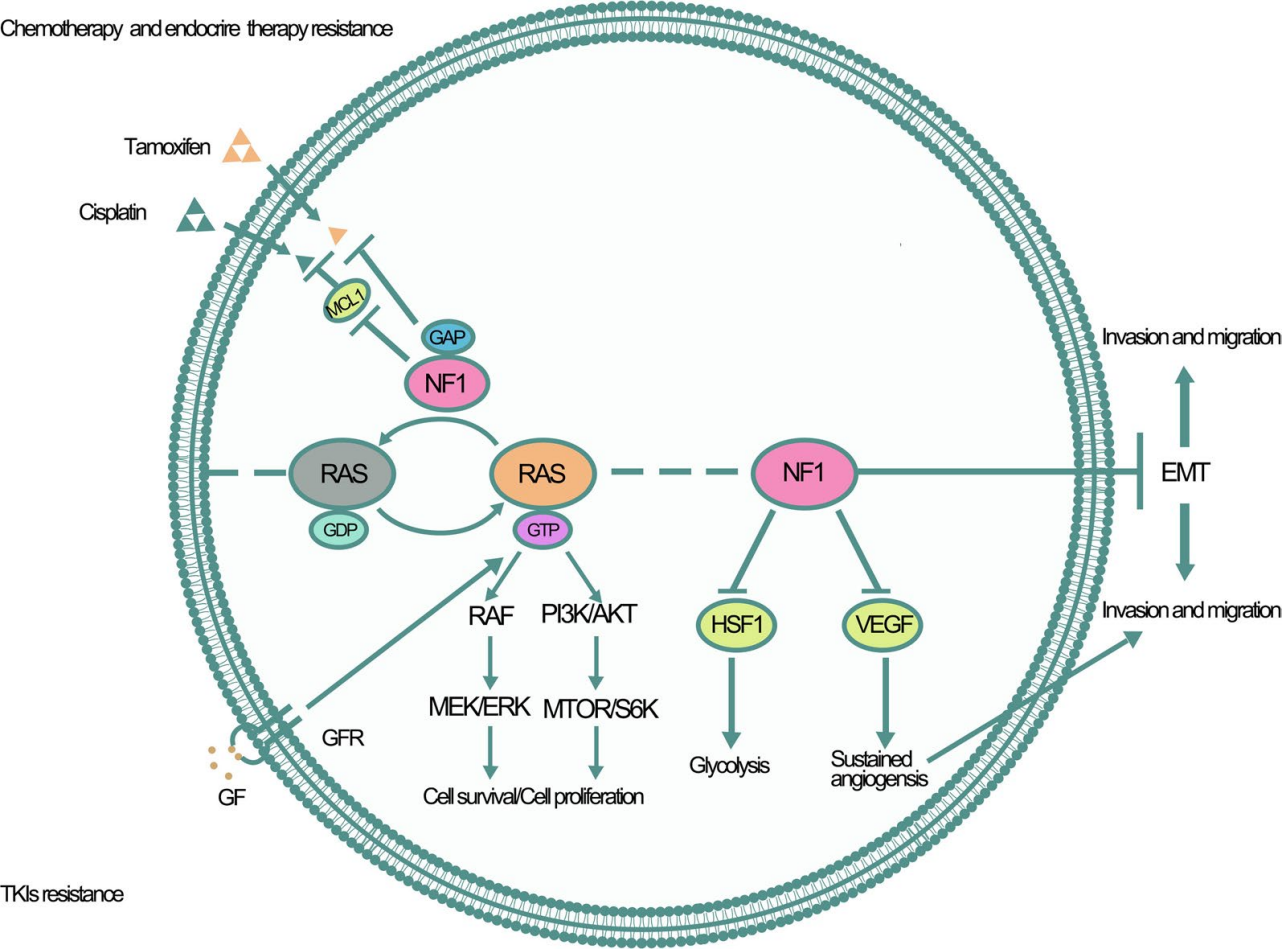
The role of NF1 in resistance to anticancer therapeutics. NF1 might be involved in resistance to anticancer therapeutics through several mechanisms, which included: NF1 downregulated MCL1 expression and endowed cells with cisplatin resistance; NF1 mutations leaded to tamoxifen acquired resistance and the mechanism was still uncertain; NF1 mutations participated in TKIs resistance via promoting glycolysis, angiogenesis, EMT, cellular proliferation and survival. GTP = guanosine triphosphate. RAS = rat sarcoma viral oncogene homologue. GDP = guanosine diphosphate. RAF = murine sarcoma viral oncogene homologue. MEK = MAPK-ERK kinase. PI3K = phosphatidylinositol-3–kinase. AKT = V-akt murine thymoma viral oncogene homologue 1. mTOR = mammalian target of rapamycin. Rac1 = Ras-related C3 botulinum toxin substrate 1. PAK1 = P21-Activated Kinase

Angiogenesis is necessary for tumor growth and metastasis, and the transition to an angiogenic phenotype depends on the result of a balance between pro-angiogenic and anti-angiogenic factor expression for most tumors [55]. Using *NF1* heterozygous mice model, Wu et al. demonstrated increased neovascularization in both the retina and cornea in response to hypoxia and bFGF, which was associated with heightened endothelial cell proliferation and migration, and increased infiltration of inflammatory cells including macrophage and mast cells [56]. Thomas et al. found that compared to normal human Schwann cells, neurofibromin deficiency was associated with the upregulation of proangiogenic factors and the downregulation of antiangiogenic factors, which enhanced the carcinogenicity of carcinogenic Schwann cells [57]. Kawachi et al. demonstrated that *NF1* gene silencing in both Schwann cells and non-Schwann cells directly leads to activation of the mTOR- hypoxia-inducible factor-1α (HIF-1α)- vascular endothelial growth factor (VEGF) pathway, which in turn increases VEGF expression [58]. Bevacizumab, a well-known VEGF inhibitor, inhibits tumor proliferation and angiogenesis through the inhibition of the VEGF pathway. A study showed that after treatment with bevacizumab, adult recurrent high-grade glioma patients harboring *NF1* mutations had prolonged postrecurrence survival [59]. Angiogenesis was proven to be related to TKI resistance [60], and *NF1* mutations might participate in the resistance to TKIs by promoting tumor angiogenesis. However, this mechanism has not been directly verified, and further research is needed. The role of *NF1* mutations in chemotherapy and TKI resistance is shown in Fig. 2.

Based on the findings above, *NF1* mutations might directly or indirectly lead to changes in several important biological behaviors of malignancies, including cell proliferation, antiapoptosis, angiogenesis, and metastasis. These behaviors are related to not only resistance to targeted therapy but also other anticancer therapeutics. This suggests that *NF1* mutations play an important role in the generation and development of malignancies and might induce resistance to anticancer therapy. The mechanisms of therapeutic resistance induced by *NF1* mutations are also summarized in Table 1.

## Novel therapies for *NF1* mutant malignancies

As a negative regulator of Ras signaling, NF1 loss results in Ras-dependent drug resistance. Previous studies have shown that the application of inhibitors of Ras and its downstream targets could overcome drug resistance induced by *NF1* mutations. Ras inhibitors, such as FTS, and mTOR inhibitors, such as everolimus, can inhibit the growth of *NF1* mutated malignancies [61, 62]. Beauchamp et al. found that for *NF1*-mutated lung adenocarcinoma resistant to dasatinib, the knockdown of ERK1/2 was sufficient to kill dasatinib-resistant cells [37]. Treatment of lung cancer with low levels of NF1 expression with MAP-ERK kinase (MEK) inhibitors can restore sensitivity to erlotinib and reverse erlotinib resistance. Therefore, concurrently using EGFR and MEK inhibitors might be superior to monotherapy for TKI-resistant *NF1*-mutated lung cancer [8]. In other studies, inhibitors of PI3K, the downstream effector of the Ras pathway, were shown to inhibit the growth of *NF1*-mutated MPNST cells and neurofibromin-deficient human breast cancer xenografts in mice [63, 64]. STAT3 is a downstream molecule of the PI3K/Akt/mTOR pathway, and natural cucurbitacin-I is a potent STAT3 inhibitor that inhibits the growth of *NF1*-mutated MPNST cells in vitro and in vivo [65].

Other therapies for *NF1* mutated malignancies, such as anti-angiogenic drugs and HSF1 inhibitors, have been reported. However, further research is still needed [46, 58, 66].

## Conclusion

In conclusion, drug resistance is a serious problem in the clinical treatment of malignancies. Understanding the mechanisms of drug resistance is critical for the development of new therapeutic strategies. According to our review, *NF1* mutations play an important role in the generation, development and drug resistance of malignancies. *NF1* mutations are involved in chemotherapy and targeted therapy resistance in tumor cells through multiple mechanisms. Inhibition of *NF1* downstream targets is an effective strategy for overcoming resistance induced by *NF1* mutations. Therefore, *NF1* mutations might be novel therapeutic targets for cancer treatment.

## Supplementary information

**Additional file** **1****: Figure S1.** The different domains of the neurofibromin protein.

## Data Availability

The data and material in this review all come from published papers.

## Abbreviations

*NF1*: Neurofibromin 1
EGFR: Epidermal growth factor receptor
EGF: Epidermal growth factor
TKI: Tyrosine kinase inhibitor
EMT: Epithelial-mesenchymal transition
HSF1: heat shock factor 1
MCL1: Myeloid cell leukemia 1
GRD: GTPase-activating protein-related domain
VEGF: Vascular endothelial growth factor
