# Supplementary figures and images for: Advancement in research and therapy of NF1 mutant malignant tumors
Source: Cancer Cell Int. 2020 Oct 9;20:492. doi: 10.1186/s12935-020-01570-8 (PMC7547409; doi:10.1186/s12935-020-01570-8)

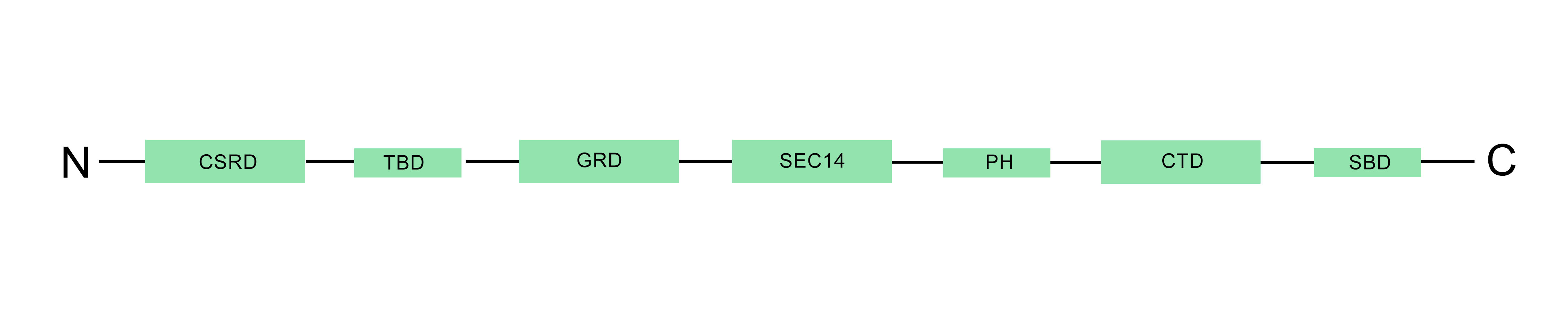

Supplement: Supplementary file 1 — Additional file 1: Figure S1. The different domains of the neurofibromin protein. [file 12935_2020_1570_MOESM1_ESM.jpg]
